# Neuropeptides SP and CGRP Diminish the *Moraxella catarrhalis* Outer Membrane Vesicle- (OMV-) Triggered Inflammatory Response of Human A549 Epithelial Cells and Neutrophils

**DOI:** 10.1155/2018/4847205

**Published:** 2018-08-05

**Authors:** Daria Augustyniak, Justyna Roszkowiak, Izabela Wiśniewska, Jacek Skała, Daiva Gorczyca, Zuzanna Drulis-Kawa

**Affiliations:** ^1^Department of Pathogen Biology and Immunology, Institute of Genetics and Microbiology, University of Wroclaw, Przybyszewskiego 63/77, 51-148 Wrocław, Poland; ^2^Department of Genetics, Institute of Genetics and Microbiology, University of Wroclaw, Przybyszewskiego 63/77, 51-148 Wrocław, Poland; ^3^3rd Department and Clinic of Paediatrics, Immunology and Rheumatology of Developmental Age, Wroclaw Medical University, ul. Koszarowa 5, 51-149 Wrocław, Poland

## Abstract

Neuropeptides such as substance P (SP) and calcitonin gene-related peptide (CGRP) play both pro- and anti-inflammatory activities and are produced during infection and inflammation. *Moraxella catarrhalis* is one of the leading infectious agents responsible for inflammatory exacerbation in chronic obstructive pulmonary disease (COPD). Since the airway inflammation in COPD is connected with activation of both epithelial cells and accumulated neutrophils, in this study we determined the *in vitro* effects of neuropeptides on the inflammatory potential of these cells in response to *M. catarrhalis* outer membrane vesicle (OMV) stimulant. The various OMV-mediated proinflammatory effects were demonstrated. Next, using hBD-2-pGL4[*luc2*] plasmid with luciferase reporter gene, SP and CGRP were shown to inhibit the IL-1*β*-dependent expression of potent neutrophil chemoattractant, hBD-2 defensin, in transfected A549 epithelial cells (type II alveolar cells) upon OMV stimulation. Both neuropeptides exerted antiapoptotic activity through rescuing a significant fraction of A549 cells from OMV-induced cell death and apoptosis. Finally, CGRP caused an impairment of specific but not azurophilic granule exocytosis from neutrophils as shown by evaluation of gelatinase-associated lipocalin (NGAL) or CD66b expression and elastase release, respectively. Concluding, these findings suggest that SP and CGRP mediate the dampening of proinflammatory action triggered by *M. catarrhalis* OMVs towards cells engaged in lung inflammation *in vitro*.

## 1. Introduction

The immunological milieu during lung inflammation may have significant implications for its resolution or progression to chronicity. The excessive inflammation is a hallmark of lower respiratory tract infections and chronic pulmonary diseases including acute respiratory distress syndrome, chronic obstructive pulmonary disease (COPD), or asthma [1]. *Moraxella catarrhalis* is one of the major infective causes of inflammatory exacerbation in COPD [2, 3]. It releases outer membrane vesicles (OMVs) decorated with a huge array of pathogen-associated molecules which can trigger inflammatory response [4]. Epithelial cells, alveolar macrophages, and neutrophils recruited into the lungs have been implicated to play an important role in the pathogenesis of COPD disease since the interplay between pivotal structural epithelial cells and inflammatory neutrophils perpetuates a state of chronic inflammation which in turn causes airway remodeling and their obstruction [1, 5, 6]. In response to common pathogens and proinflammatory cytokines such as IL-1*β* and TNF-*α*, the activated pulmonary epithelial cells, including type II alveolar cells, produce an array of inflammatory chemokines such as IL-8, MCP-1, and human *β*-defensin hBD-2. These chemokines help to attract the inflammatory leukocytes including neutrophils to the lungs [7–10]. Neutrophils also contain a huge array of inflammatory mediators stored in azurophilic and specific granules which are released outside following cellular activation [11]. Furthermore, the proapoptotic activity of various bacterial stimuli exerted on epithelial lining together with the huge oxidative stress associated with neutrophil degranulation belongs to other factors that may escalate inflammation of the respiratory tract [12]. Interestingly, in the distal airways of patients with COPD and acute respiratory failure, high levels of hBD-2 are correlated with increased neutrophil survival [13].

Numerous endogenous agents have been verified as agents able to protect cells from damage caused by harmful inflammation. Neuropeptides calcitonin gene-related peptide (CGRP) and substance P (SP) have been proposed as one of them. In the respiratory tract, CGRP and SP are expressed by nerve fibers projecting to the airways and by pulmonary neuroendocrine cells of the airway epithelium [14, 15]. Furthermore, these neuropeptides can be produced by immune cells such as dendritic cells, monocytes, neutrophils, and lymphocytes [16, 17]. Both CGRP and SP serve as regulators of numerous airway functions including bronchoconstriction of airway smooth muscles and vasodilation of airway vessels [18, 19]. Both neuropeptides have direct and indirect antimicrobial, mainly innate immunomodulatory properties [17, 20]. Numerous studies have shown that these neuropeptides released at sites of inflammation may play proinflammatory activities mainly through intensification of IL-1*β*, IL-6, and TNF-*α* expression [18, 19, 21, 22]. Nevertheless, both neuropeptides may exert potent anti-inflammatory effects as well. The most essential of them is SP involvement in tissue repair by the promotion of growth of fibroblasts and endothelial cells [19] or by the induction of transition from proinflammatory macrophages into M2-like macrophages responsible for tissue repair [23]. CGRP, in turn, as one of the most potent anti-inflammatory neuropeptides, can directly act on macrophages and dendritic cells, thus inhibiting their capacity to produce inflammatory cytokines. This effect of CGRP is mainly due to upregulation of the immunosuppressive cytokine IL-10 and inhibition of antigen presentation to T cells [24–26]. Likewise, CGRP attenuates IL-1*β*-induced MCP-1 secretion and suppresses the IL-1*β*-evoked ROS-NF-*κ*B cascade [27]. It suppresses also hyperoxia-induced oxidative stress-related injury to primary alveolar epithelial type II cells [28]. We have previously shown anti-inflammatory activity of CGRP by modulating the phagocytic function of human neutrophils and enhancing the direct antibacterial effects of these cells [29].

No study has addressed the beneficial anti-inflammatory role of SP and CGRP in reduction of inflammatory and proapoptotic potential triggered by *M. catarrhalis* OMVs towards the A549 airway epithelium, and no data are available on their influence on OMV-induced neutrophil granule exocytosis. Therefore, the present study was designed to elucidate the impact of both neuropeptides on (i) OMV-stimulated hBD-2 promoter activity in human A549 epithelial cells (type II alveolar cells) as constituents of the first line of defense, (ii) OMV-mediated A549 apoptotic response, and (iii) the azurophilic and specific granule release from neutrophils—the processes associated with the damage of surrounding tissues.

## 2. Materials and Methods

### 2.1. Reagents

Cytochalasin D, dextran, DMSO, fMLP (*N-*formyl-methionyl-leucyl-phenylalanine), MTT (thiazolyl blue tetrazolium bromide), and trypan blue were from Sigma, Germany; human neutrophil elastase and elastase fluorogenic substrate MeoSuc-Ala-Ala-Pro-Val-AMC were from Calbiochem (Germany); antibiotic-antimycotic solution (100x) containing 10,000 units/ml of penicillin, 10,000 *μ*g/ml of streptomycin, and 25 *μ*g/ml of Fungizone, GlutaMAX (100x), heat-inactivated fetal bovine serum (HiFBS), and sodium pyruvate (100x) were from Gibco Life Technologies; DMEM, RPMI-1640 media, and trypsin-EDTA solution were from Lonza (Belgium), and rhIL-1*β* from R&D; pGL4.10[*luc2*] vector, TransFast Transfection Reagent E2431, and luciferase assay system were from Promega (USA); calcitonin gene-related peptide (CGRP-*α*) and substance P were from Tocris Bioscience (USA); NGAL DuoSet ELISA and TMB substrate reagent were from R&D Minneapolis (USA); QIAamp DNA Mini Kit, MinElute PCR Purification Kit (Qiagen), Invisorb Spin DNA Extraction kit (Invitek), and TOPO TA Cloning Kit were from Invitrogen (Thermo Fisher Scientific). *High*-*Fidelity* and *Taq* polymerases as well as restriction enzymes: Pst I, Kpn I, and Hind III were from Fermentas (Thermo Fisher Scientific). Antibodies CEACAM1 mAb (283340), goat anti-mouse IgG (H + L), superclonal secondary antibody conjugated to Alexa Fluor 488, CD66b mAb (G10F5) conjugated to FITC, and mouse IgM isotype control conjugated to FITC were from Invitrogen, Thermo Fisher Scientific.

### 2.2. Cell Line Culture Condition

The A549 human epithelial cell line (type II alveolar cells, ATCC CCL-185) was cultured in DMEM medium supplemented with 10% HiFBS, 1x GlutaMAX, and 1x antibiotic-antimycotic solution at 37°C in the presence of 5% CO_2_. To obtain a fully confluent monolayer, cells were grown for 2–3 days. Before a new passage, cells were trypsinized with trypsin-EDTA solution and washed with DMEM. The line was propagated in flasks or microplates from Nunc (Thermo Fisher Scientific).

### 2.3. Isolation of Neutrophils

Heparinized venous blood was obtained from healthy volunteers, and the responsible Ethical Committee has approved these experiments in accordance with the Declaration of Helsinki (1964). Neutrophils were isolated by dextran sedimentation followed by centrifugation over discontinuous plasma-Percoll gradients. Percoll gradient in 0.9% NaCl was composed of 1.5 ml of 61% Percoll which was underlayered by 1.5 ml of 76% Percoll. Heparinized peripheral venous blood was gently mixed with PBS buffer (pH 7.4) containing 2% dextran in a 1 : 1 ratio. The cell suspension was left at room temperature for erythrocyte sedimentation to occur. The leukocyte-rich plasma (3–6 ml) was carefully transferred to Percoll gradient and centrifuged (550 ×g/30 min). Subsequently, the PMN band (≥95% neutrophils) at the interface of the 61% and 76% Percoll layers was collected and transferred to a 15 ml falcon tube followed by hypotonic lysis of erythrocytes with a lysing buffer (150 mM NH_4_Cl, 10 mM KHCO_3_, and 0.3 mM EDTA, pH 7.4). After two washes (320 × g/10 min) in PBS, neutrophils were suspended in RPMI without antibiotics and kept for 30 min at 37°C and 5% CO_2_ until used. Cells were assessed for viability with the trypan blue exclusion assay.

### 2.4. Outer Membrane Vesicle Isolation

Outer membrane vesicles (OMVs) were isolated as reported previously [4] with some modifications. Briefly, 18 h cultures of *M. catarrhalis* strains were diluted 50-fold in 500 ml of brain-heart infusion (BHI) broth and incubated at 37°C for 16–18 h with orbital shaking (150 rpm). The cultures were centrifuged at 6600 ×g for 15 min at 4°C. The supernatants were collected and passed through a 0.22 *μ*m pore-size filter vacuum pump (Merck, Millipore). The filtrates were concentrated using 50 or 100 kDa Vivaspin centrifugal concentrators (Amicon Ultra, Millipore) at 5000 ×g for 30 min at 4°C. The concentrated supernatants were subsequently pelleted overnight (100,000 ×g, at 4°C) in an ultracentrifuge (Beckman Coulter Optima). The pelleted OMVs were resuspended in 500 *μ*l of sterile PBS buffer (pH 7.4), aliquoted, and stored at −20°C. The sterility of OMV preparations was confirmed on BHI agar. The protein concentrations in OMV preparations were measured using a Qubit fluorometer or Bradford assay (Sigma-Aldrich Inc.), and the quality of OMV preparations was confirmed in 12% SDS-PAGE.

### 2.5. TEM

18 h cell culture of *M. catarrhalis* in BHI was centrifuged and rinsed in PBS. The pellet was fixed in 1 ml of cacodylate buffer (0.2 M sodium cacodylate, 0.2 M HCl, pH 7.4) supplemented with 2.5% glutaraldehyde and incubated 8–10 h at room temperature (RT). The suspension was rinsed by centrifugation (3000 ×g, 10 min, RT) several times with cacodylate buffer. The resultant pellet was postfixed in cacodylate buffer containing 1% OsO_4_ for 2 h at RT and rinsed. The samples were subsequently dehydrated in a series of ethanol concentrations and embedded in Epon 812. Thin sections were cut with an ultramicrotome (Reichert-Jung) equipped with a diamond knife and stained with 2% uranyl acetate and lead citrate. The samples were then visualized with a TEM (TESLA BS 540) operated at 80 kV.

Isolated OMVs were visualized by negative staining. Briefly, a drop of a PBS-diluted suspension of the OMVs (~0.075 mg/ml) was placed on a 200-mesh Formvar copper grid (Christine Gröpl Electronenmikroskopie, Austria), allowed to adsorb, and the surplus was removed by filter paper. A drop of 2% (*w*/*v*) aqueous solution of uranyl acetate was added and left in contact with the sample for 5 minutes and dried. The OMVs were imaged with a TEM operating at an acceleration voltage of 150 KV (Hitachi H-800).

### 2.6. hBD-2 Reporter Plasmid Construction

The generation of reporter construct of the human *β*-defensin 2 promoter was adapted from previously published methods [30, 31]. The hBD-2 promoter sequence was amplified from PstI-digested genomic DNA by polymerase chain reaction (PCR) using primers 5′-GAG GTA CCT CCA TCC TTT ACT GTG ATG ATG CC-3′ (forward primer with Kpn1 tail), 5′-GAA AGC TTT GGC TGA TGG CTG GGA GCT TCA CCA (reverse primer with HindIII tail), and *Fidelity* polymerase. The hBD-2 gene accession number was NG_023301.1. The amplified product (2.6 kbp) was subcloned into the TOPO vector, and after electroporation of *E. coli* Mach1-T1, the relevant insert was subcloned into the multiple cloning site of the pGL4.10[*luc2*] vector to generate hBD-2-pGL4[*luc2*] luciferase plasmid. The identity of the subcloned reporter construct of the *β*-defensin 2 promoter was confirmed by DNA sequencing (GenoMed, Warsaw, Poland).

### 2.7. Transfection and Luciferase Assay

All transient transfections were carried out in triplicate using TransFast Transfection reagent E2431 according to the manufacturer's instructions. A549 cells were seeded into 96-well plates at a density of 10^4^ cells/well and were transfected with hBD-2-pGL4[*luc2*] plasmid or empty vector upon reaching 60–70% confluence. The transfected cells were not selected with antibiotics. OMVs, IL-1*α*, and neuropeptides were added to the transfected cells 24 h post-transfection, and the exposition time was 8 hr in the presence of HiFBS. All transfections were carried out in duplicate. The cells were then washed with phosphate-buffered saline, dissolved in 200 *μ*l of cell culture lysis reagent, and harvested. To analyze hBD-2 promoter activity, luciferase activity was measured using a luminometer (Varioskan™ Flash Multimode Reader, Thermo Fisher Scientific) after adding luciferase substrate, according to the manufacturer's instructions. Promoter activities were expressed in relative light units RLU/s as fold increase of luciferase activity, compared to nonstimulated controls. Plasmid pGL4.13 [luc2/SV40] with constitutive expression of the luciferase promoter was used as positive control of transfection.

### 2.8. OMV Cytotoxicity

- For the cell viability assessment, A549 cells were seeded at an initial density of 2 × 10^4^ cells per well at a final volume of 100 *μ*l in 96-well flat-bottom microplates (NUNC) and incubated under 5% CO_2_ at 37°C for 24 h. The various concentrations of Mc6 OMVs at 100 *μ*l volume were added for the next 8 or 24 h. Cell viability was determined by the MTT colorimetric assay as described previously [32]. Briefly, four hours before the end of incubation, 20 *μ*l MTT (5 mg/ml) was added to each well. The formazan produced was solubilized with 180 ml of dimethyl sulfoxide for 15 min with shaking. After addition of 12.5 ml of Sørensen's phosphate buffer (pH 7.2), the concentration of formazan was determined by optical density measurement at 570 nm using the Asys UVM 340 spectrophotometer (Biochrom, Cambridge, UK). The cytotoxic activity of the studied compounds was expressed as inhibition of proliferation rate of treated cells compared with control untreated cells.
- For apoptosis evaluation, 0.5 ml of A549 cells was cultured in DMEM medium in 24-well microplates at initial 4 × 10^5^ cells/ml density for 18 hours. The cell monolayer was treated with OMVs at different concentrations in the presence or absence of physiologic concentrations of neuropeptides for 8 and 24 h. After treatment, the cells were trypsinized, harvested, and washed with ice-cold PBS. The frequencies of early apoptotic, late apoptotic, and necrotic cells were evaluated with “Annexin V & Dead Cell Assay kit” according to the manufacturer's instructions (Merck Millipore, Darmstadt, Germany). This assay is based on phosphatidylserine (PS) detection on the apoptotic cell surface, using fluorescently labeled Annexin V in combination with the dead cell marker, 7-aminoactinomycin D (7-AAD). The apoptotic ratio has been calculated by identifying four populations: (i) viable cells, not undergoing detectable apoptosis: (i) Annexin V (−) and dead cell marker (−), (ii) early apoptotic cells: Annexin V (+) and dead cell marker (−), (iii) late apoptotic cells: Annexin V (+) and dead cell marker (+), and (iv) cells that died through the nonapoptotic pathway: Annexin V (−) and dead cell marker. The fluorescence was determined using a Muse Cell Analyzer (Merck Millipore). Data for 2000 events were stored. The Muse computer system software was used for data acquisition and analysis. In some cases, the washed cells were stained for flow cytometry analysis.

### 2.9. Neutrophil Degranulation Assay

The human neutrophils were degranulated as previously described [33]. To test the effect of OMVs and NPs on resting human neutrophils, 1 × 10^6^ cells/ml were gently mixed and 500 *μ*l was aliquoted into polystyrene cell culture tubes. To obtain degranulation, all samples (without spontaneous degranulation sample) were primed with cytochalasin D (5 *μ*g/ml) for 5 min at 37°C, 5% CO_2_ followed by a further 30 min incubation (37°C, 5% CO_2_) with stimulants including OMVs, neuropeptides, and in some experiments *M. catarrhalis* in different combinations. Positive degranulation controls were performed by stimulating the cells with 1 *μ*M fMLP. After incubation, cells were centrifuged (250 ×g for 5 min) and cell-free supernatants were collected and stored at −20°C until being assayed for elastase and NGAL as markers of azurophilic and specific granule content, respectively. In some cases, pelleted neutrophils were washed, resuspended in 100 *μ*l HBSS buffer, and stained for flow cytometry analysis.

### 2.10. Granule Marker Assessment

Neutrophil elastase (NE) activity was determined by measuring the cleavage of the fluorogenic NE substrate, MeoSuc-Ala-Ala-Pro-Val-AMC, dissolved in Hank's Balanced Salt Solution (HBSS) reaction buffer at pH 7.5, containing 0.1% (*w*/*v*) HEPES, 10% (*v*/*v*) DMSO, and 150 mM NaCl as determined previously [33, 34]. The working substrate concentration that gave the linear relationship (increase in fluorescence) was 100 *μ*M as determined in preliminary calibration curve experiments with various concentrations of elastase. Cell-free suparnatants after degranulation were added to the substrate in a 1 : 1 ratio in a volume of 50 *μ*l each and were immediately measured using the 96-well flat-bottom black microplate (NUNC). The cleavage rate of the substrate measured for 30 min at 37°C as the increase in fluorescence was monitored spetrofluorometrically (Varioskan™ Flash Multimode Reader, Thermo Scientific) at excitation wave *λ* = 370 nm and emission *λ* = 445 nm.

NGAL concentrations were assayed using DuoSet ELISA Development using TMB as a substrate.

### 2.11. Flow Cytometric Analysis

A549 cells in 200 *μ*l PBS buffer (ap. 5 × 10^5^ cells) were stained with mouse anti-CEACAM1 (clone 283340) primary mAbs (5 *μ*g/ml), diluted in 2% HiFBS/PBS for 1 h on ice, washed, and incubated with Alexa Fluor 488-conjugated goat anti-mouse IgG superclonal secondary antibody (1 *μ*g/ml) for 0.5 h on ice followed by washing. Neutrophils (ap. 5 × 10^5^ cells) were stained with FITC-conjugated anti-CD66b mAbs IgM (0.5 *μ*g per test). Background fluorescence was determined using isotype matched control. Fluorescence was measured by FACSCalibur (Becton Dickinson) collecting 10,000 events. Data were obtained using Becton Dickinson software, and further analysis was performed using WinMDI 2.8 software.

### 2.12. Statistical Analysis

The data were expressed as the mean ± SD, and analyzed for significant difference by one-way ANOVA followed by Tukey's post hoc tests test using the Statistica (version 13.1) software. Differences were considered statistically significant if *p* < 0.05.

## 3. Results

### 3.1. Secretion of OMVs from *M. catarrhalis* Mc6

**Figure 1:**
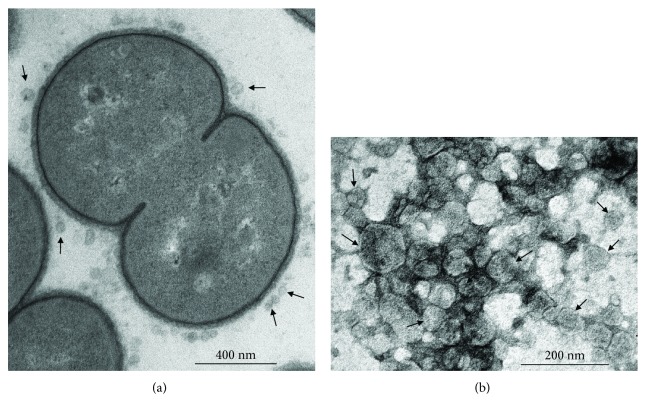
Transmission electron microscopy (TEM) micrographs of *Moraxella catarrhalis* (Mc6) outer membrane vesicles (OMVs). (a) Bacteria releasing OMVs. Bacteria were harvested from *in vitro* overnight cultures and prepared as described in Materials and Methods. Shown are electron micrographs of single cells (magnification, ×22,000). (b) The isolated OMVs (magnification, ×50,000). OMVs were examined by TEM as described in Materials and Methods. Shown micrographs are illustrative of at least two independent experiments. The released vesicles are indicated by arrows.

We first analyzed the secreted OMVs from *M. catarrhalis* Mc6 clinical strain (GenBank accession number: CP010901) during *in vitro* culture in BHI broth. Transmission electron microscopy (TEM) demonstrated that *M. catarrhalis* secreted 30–120 nm spherical vesicles into the extracellular milieu (Figure 1(a)). The OMVs were collected from the culture supernatants following ultrafiltration and ultracentrifugation (Figure 1(b)). The proteomic analysis of Mc6 OMVs conducted by LC-MS/MS spectrometry revealed that pivotal outer membrane proteins packaged in these vesicles were OmpCD, OmpE, UspA1, Hag/MID, CopB, MhuA, TbpA, TbpB, LbpB, OMP M35, and MipA [35]. Mc6 OMVs contained LOS serotype A as determined previously [36]. Summing up, Mc6 OMVs harbor potentially active virulence factors, which can perform diverse biological functions in host cells.

### 3.2. *M. catarrhalis* OMVs Activate hBD-2 Promoter in A549 Epithelial Cells

**Figure 2:**
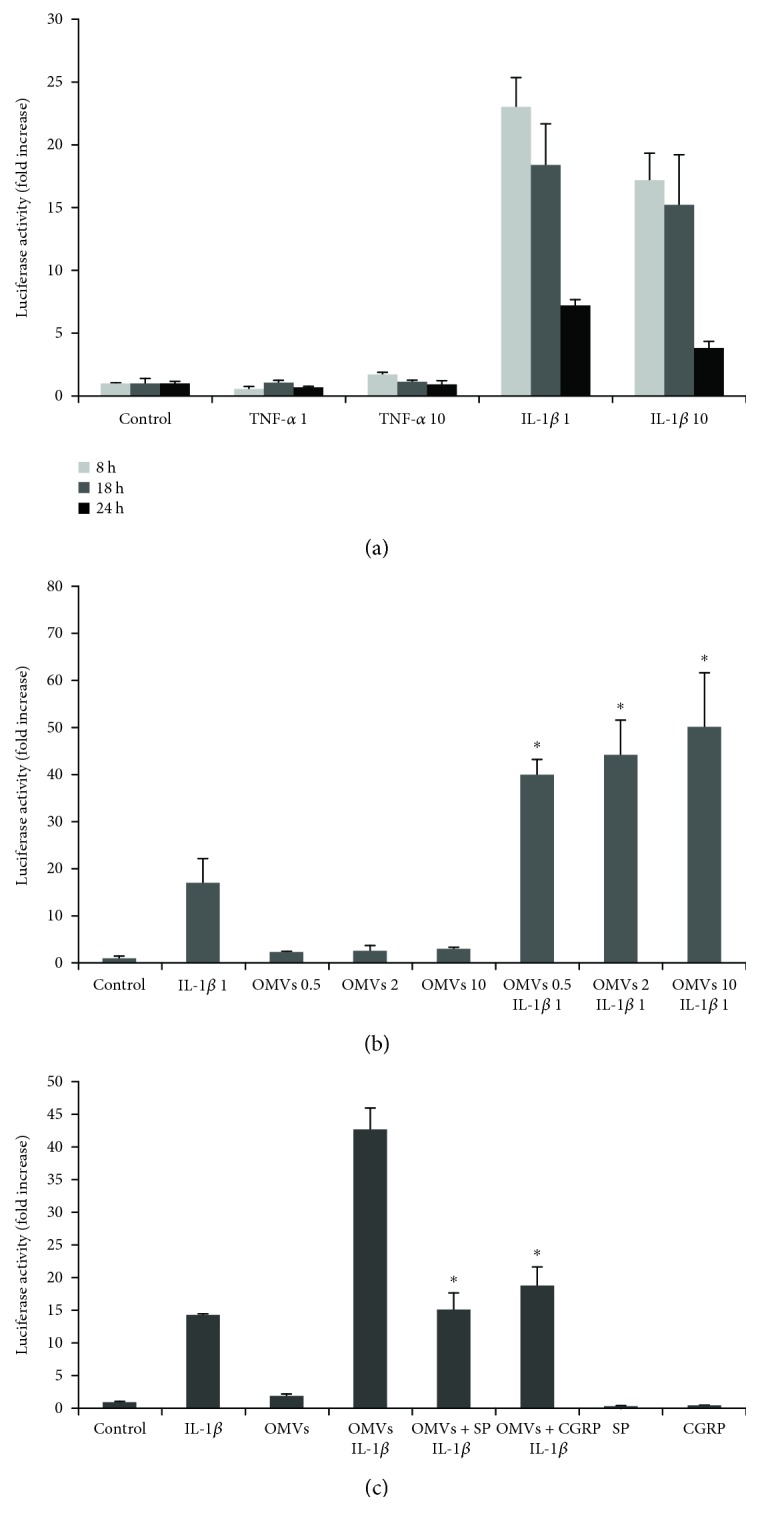
Modulation of hBD-2 promoter activity in human lung epithelial cells A549 by *M. catarrhalis* outer membrane vesicles (OMVs) and neuropeptides. Stimulant concentration: (a) the indicated cytokine concentrations are given in ng/ml, (b) the indicated concentrations of OMVs are given in *μ*g/ml, whereas IL-1*β* in ng/ml (c) stimulants were used at the following concentrations: IL-1*β* (1 ng/ml), OMVs (2 *μ*g/ml), SP, and CGRP (10^−8^ M). The cell line was transfected with luciferase reporter vector containing the hBD-2 promoter (hBD-2-pGL4[*luc2*]) and incubated with stimulants. (a) Kinetics of cytokine-dependent hBD-2 expression. (b) IL-1*β*-induced hBD-2 expression in cells stimulated by *M. catarrhalis* OMVs; ^∗^*p* < 0.001 compared with cells stimulated only with IL-1*β* or the corresponding concentrations of OMVs alone. (c) Neuropeptide-dependent decrease of IL-1*β*-induced hBD-2 expression in cells stimulated by *M. catarrhalis* OMVs; ^∗^*p* < 0.001 compared with cells stimulated simultaneously with OMVs and IL-1*β*. Data are expressed as the *n*-fold increase in luciferase activity measured in RLU/s relative to unstimulated control. Error bars indicate the SEM of the mean values obtained from two (a, b) and at least three (c) independent experiments performed in duplicate. Differences between luciferase activities were analyzed by one-way ANOVA followed by Tukey's post hoc test.

Human beta defensin 2 (hBD-2) can reach significant levels in bronchial alveolar lavage fluid in COPD patients [8] and is modulated mostly by IL-1*β* [31, 37]. There are no data regarding the role of *M. catarrhalis* in activation of hBD-2 defensin from epithelial cells. In preliminary kinetic experiments, we confirmed that hBD-2 promoter activity in transfected A549 cells was induced by IL-1*β* in a time- and dose-dependent manner with the strongest hBD-2 response after 8 h of induction in regards to the studied time framework (Figure 2(a)). The induction of hBD-2 by TNF-*α* was much lower, and there were no synergistic actions between both cytokines in hBD-2 activation (data not shown). Next, after 8 h of incubation, we examined the contribution of IL-1*β* to OMV-stimulated activation of the hBD-2 promoter. As shown in Figure 2(b), the stimulation of A549 by OMVs in the range from 0.5 to 10 *μ*g/ml (protein concentration) caused only weak activation of hBD-2 promoter activity. In contrast, when OMVs were used in combination with IL-1*β*, an over 40-fold increase in hBD-2 promoter activity was observed in comparison to untreated control that gave a near 3-fold stronger activation of hBD-2 than free IL-1*β*. We did not test the higher concentrations of OMVs because, as we documented, they caused apoptotic cell death of A549 cells. These results indicate that *M. catarrhalis* OMVs significantly activate the hBD-2 promoter in A549 cells in the presence of IL-1*β*.

### 3.3. Neuropeptides SP and CGRP Inhibit *M. catarrhalis* OMV-Stimulated hBD-2 Expression by A549

To further assess the effect of SP and CGRP on IL-1*β*-induced hBD-2 expression in response to *M. catarrhalis* OMVs, A549 cells were treated with 2 *μ*g/ml of OMVs simultaneously with physiological concentrations of both neuropeptides (10^−8^ M) for 8 hours. As shown in Figure 2(c), the exogenously added SP or CGRP markedly reduced the IL-1*β*-induced hBD-2 expression in cells stimulated by OMVs causing a 3-fold and 2.3-fold decrease in promoter activity, respectively. Both neuropeptides used alone failed to activate the hBD-2 promoter. These results suggest that neuropeptides SP and CGRP are involved in the inhibition of hBD-2 promoter activity in A549 cells in response to *M. catarrhalis* OMVs and IL-1*β* stimulators. In particular, the stronger inhibitory effect of SP indicates a more important role of this neuropeptide in the observed phenomenon.

### 3.4. Neuropeptides SP and CGRP Rescue A549 Cells from OMV-Induced Cell Death and Apoptosis

**Figure 3:**
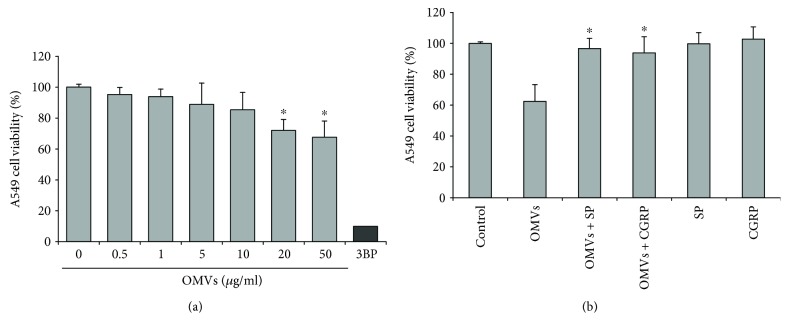
Neuropeptide-mediated abrogation of cytotoxic effect of *M. catarrhalis* OMVs on A549 human epithelial cells. The confluent cells were exposed to the indicated concentrations of OMVs (protein concentration) over 24 h of incubation, and the cell viability (% of untreated control) was determined using the MTT assay. (a) Cytotoxic effect of indicated OMVs (protein concentration) and 400 *μ*M 3-BP (3-bromopyruvate) as positive control. (b) Anticytotoxic effect of physiological concentrations (10^−8^ M) of neuropeptides towards cell death induced by OMVs at 100 *μ*g/ml. Data are expressed as the mean ± SD of three independent experiments performed at least in four replicates. Statistics was performed by one-way ANOVA followed by Tukey's post hoc test. (a) ^∗^*p* < 0.001 compared to untreated control and (b) ^∗^*p* < 0.001 compared to OMV treatment.

The inflammatory response is accompanied by excessive apoptosis of cells engaged in inflammation. Since *M. catarrhalis* OMVs can induce inflammatory response of A549, the next step was to evaluate the viability of A549 cells treated with *M. catarrhalis* OMVs. Cells were treated with various concentrations of OMVs for 8 h and 24 h, and MTT assay was performed. After 8 h of incubation, the cytotoxic effect of OMVs was negligible (data not shown). As presented in Figure 3(a), the exposure of A549 to OMVs in the range of 1–10 *μ*g/ml over the 24 h did not modify the viability of the cells. OMVs used at concentration of 20 *μ*g/ml and higher showed a considerable increase in cytotoxicity. We next exposed A549 cells to 100 *μ*g/ml OMVs for 24 h with and without physiological concentrations (10^−8^ M) of NPs. Both SP and CGRP significantly inhibited OMV-induced cell death (Figure 3(b)). In the absence of OMVs, both NPs slightly increased cell proliferation.

**Figure 4:**
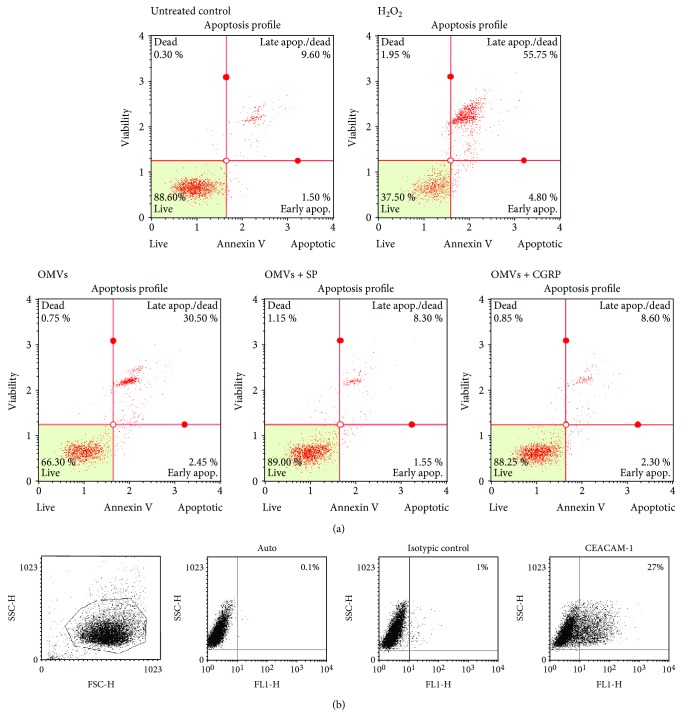
Flow cytometric analysis of antiapoptotic effects of SP and CGRP towards A549 cell death induced by *M. catarrhalis* OMVs. (a) The A549 cells were treated with various stimulants for 24 h of incubation, processed, stained with Annexin V and PI antibodies, and analyzed using Muse Cell Analyzer (Merck). The upper panel refers to untreated control cells and positive control (5 mM H_2_O_2_). The lower panel refers to the cells treated with OMVs 100 *μ*g/ml alone or in the presence of 10^−8^ M of SP or CGRP. Representative data from at least two independent experiments are shown. (b) Cell surface expression of CEACAM1 in A549 obtained from 1-day postconfluent phase of growth. A549 cells were stained for CEACAM1 with mAb clone 283340 as primary antibody or isotype-matched control followed by Alexa Fluor 488-conjugated goat anti-mouse IgG superclonal secondary antibody. Subsequently, samples were measured by FACs. The data shown are representative of two independent but essentially the same experiments performed in duplicates.

Because the MTT assay cannot distinguish cell death caused by apoptosis or necrosis, in the next step apoptotic response was established. A549 cells were exposed to 20 or 100 *μ*g/ml of OMVs for 8 h and 24 h of incubation. As shown in Table 1, the cytometric analysis demonstrated no significant cell death at 20 *μ*g/ml of OMVs at two studied time points in comparison to untreated control. In contrast, the significant differences in the frequencies of total apoptotic cells (early apoptotic and late apoptotic/necrotic) between cells stimulated with OMVs at a concentration of 100 *μ*g/ml and control were observed both after 8 h (*p* = 0.08) and 24 h (*p* < 0.005) of culture. Comparing untreated control to OMV-treated cells, the number of total apoptotic cells increased twice after 8 h and three times after 24 h. For the latter time point, the signal of the OMV*-*induced apoptosis was twice lower than the maximal signal obtained for positive control (5 mM H_2_O_2_). To invest the ability of both SP and CGRP to inhibit OMV-mediated apoptosis, A549 cells were treated over 24 h simultaneously with OMVs and physiological concentrations (10^−8^) of neuropeptides. As presented in Figure 4, SP and CGRP abolished the apoptotic effects exerted by *M. catarrhalis* OMVs with *p* < 0.001. There were no significant differences between SP and CGRP in reference to antiapoptotic activity as well as to incubation of cells in the presence of particular neuropeptides alone. Our data indicates that both SP and CGRP can rescue a fraction of A549 cells from *M. catarrhalis* OMV-induced apoptosis. The expression of CEACAM1, a receptor engaged in *M. catarrhalis-*dependent apoptotic effects [38], on 1-day postconfluent A549 cells (29% ± 4.35; mean ± SD; *n* = 2) was confirmed by flow cytometry (Figure 4(b)). OMV treatment substantially did not change CEACAM1 expression (data not shown).

### 3.5. Neuropeptides SP and CGRP Partially Inhibit the Release of Granules from OMV-Triggered Neutrophils

**Figure 5:**
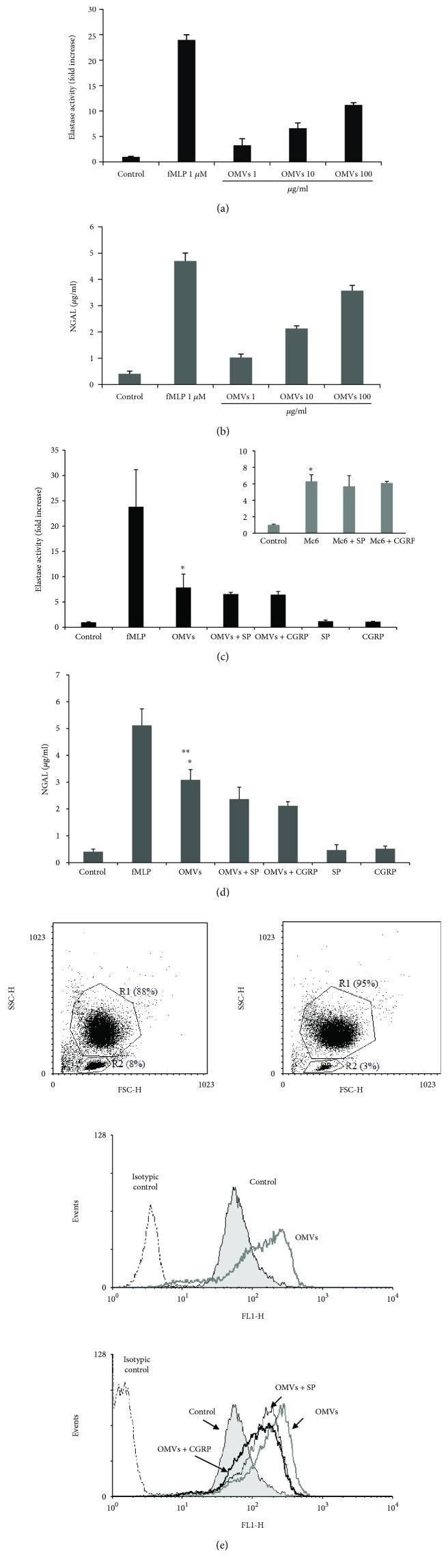
Modulation of neutrophil degranulation triggered by OMVs from *M. catarrhalis* strain Mc6 and neuropeptides. Human neutrophils at ~1 × 10^7^ cell/ml were primed with cytochalasin D (5 *μ*g/ml), incubated for 30 min with various stimulants, and assayed for the release of azurophilic (a, c) and specific (b, d, e) granule markers. The release of elastase, NGAL, and CD66b expression was assessed as described in Materials and Methods. (a, b) The dose-dependent increase in granule content release upon stimulation with OMVs. Azurophilic (c) and specific (d, e) granule release triggered by stimulants in concentrations: OMVs (50 *μ*g/ml), SP and CGRP (10^−8^ M), and fMLP (1 *μ*M) as positive control. Data in the inset of (c) performed with cells stimulated with whole bacteria Mc6 (MOI 5). (e) CD66b cell surface expression on neutrophils after activation by various stimuli, measured by flow cytometry. The panels show (upper panel) the representative FACs dot plots of analyzed cells (from two donors) after priming followed by 30 min incubation with medium alone (R1 gate refers to neutrophils, and R2 gate refers to lymphocytes and other debris) and (middle and lower panel) representative histograms of gated neutrophils after treatments. Cells were stained for CD66b with FITC-conjugated mAb or isotype-matched control and measured. FACs histograms are representative of two independent experiments performed in duplicates. Data for azurophilic degranulation are expressed as the *n*-fold increase in elastase activity measured in RLU/s relative to unstimulated control. Error bars indicate the SD of the mean values obtained from one representative (a, b) and at least two (c, d) independent experiments performed in duplicates on neutrophils from different donors. Asterisks ^∗^ in (c) and (d) denote values significantly different from the control (*p* < 0.001). Asterisks ^∗∗^ in (d) denote values significantly different from OMVs samples treated with CGRP (*p* < 0.05). Differences were analyzed by one-way ANOVA followed by Tukey's post hoc test.

To determine whether the interaction of neutrophils with *M. catarrhalis* OMVs results in an increased degranulation of neutrophils, these cells obtained from healthy volunteers after priming with cytochalasin D were incubated for 30 min with different doses of OMVs and granular markers were evaluated in gained supernates. Neutrophil degranulation was measured by quantitating the release of elastase, an enzyme present in azurophilic granules and NGAL as the specific granule marker. As positive control, fMLP was used which caused a robust and almost complete neutrophil degranulation. As shown in Figures 5(a) and 5(b), OMV Mc6 triggered degranulation of both azurophilic and specific granules of neutrophils in a concentration-dependent manner. It indicates that *M. catarrhalis* OMVs are potent inductors of inflammatory mediator release from neutrophil granules.

The experiments were next carried out to determine whether studied neuropeptides used in physiological concentrations can decrease the degranulation of neutrophils in response to this bacterial stimulant. As shown in Figure 5(c), both SP and CGRP did not inhibit the OMV-induced neutrophils to release the azurophilic granules. In contrast, the release of specific granules was significantly decreased in the presence of CGRP (Figure 5(d)). There was no analogous significant inhibitory action of SP; nevertheless, the tendency of this neuropeptide to reduce OMV-potentiated granule exocytosis was noticed. These results were next confirmed by flow cytometry by analyzing another marker of secondary granule exocytosis, namely, CD66b. As shown in Figure 5(e) (lower panel), CD66b expression, markedly potentiated after OMV exposure (MFI ± SD; 157 ± 4.24; *n* = 2), was impaired by 30% only in the presence of CGRP (MFI ± SD; 111 ± 1.42; *n* = 2) whereas in the presence of SP this impairment was ~20% (MFI ± SD; 123 ± 2.84; *n* = 2). No apparent release of both types of granules was observed by treating neutrophils with CGRP or SP alone. The differences in degranulation potency between SP and CGRP were not observed. In conclusion, these studies suggest that treatment of neutrophils with physiological concentrations of SP and CGRP caused the partial dampening of degranulation of these cells upon *M. catarrhalis* OMV stimulation.

## 4. Discussion

The immunological milieu during airway inflammation may have significant implications for its resolution or progression to chronicity. The role of neuroendocrine mediators such as SP and CGRP in inflammatory response is still being elucidated as they seem to play both pro- and anti-inflammatory activities. Furthermore, local and systemic levels of neuropeptides increase in response to microbial infections [39]. Since the bacteria-triggered airway inflammation in COPD is strongly associated with activation of both epithelial cells and neutrophils in the lungs, in this study we determined *in vitro* the inhibitory effect of SP and CGRP on the inflammatory potential of these cells in response to *M. catarrhalis* OMVs. The OMV stimulant was chosen as it (i) is released during *M. catarrhalis* infection *in vivo* [40], (ii) contains a cargo composed of pivotal virulence factors with proinflammatory potential exemplified by OMV-mediated IL-8 and ICAM-1 expression in A549 [4], and (iii) contains LPS which may contribute to the progressive deterioration of lung function [41].

In the present study, we have demonstrated that *M. catarrhalis* OMVs in the presence of IL-1*β* triggered the prominent expression of potent neutrophil chemoattractant hBD-2 in transfected A549 cells. It indicates that OMVs may affect hBD-2-mediated pulmonary defense mechanisms. The binding of OMVs with A549 epithelial cells may have occurred by lipid raft-dependent interactions, as previously described for *M. catarrhalis* vesicles [4]. We also determined that hBD-2 expression was significantly diminished following simultaneous exposure of cells to OMVs and importantly physiological concentrations of SP or CGRP. Considering the chemotactic activity of hBD-2 toward human neutrophils [9], the hypothetical significance of our finding may propose a beneficial route of action where studied neuropeptides, by modulating the hBD-2 level, may indirectly decrease the influx of neutrophils to the lungs. This may also offer a negative response as the hBD-2 peptide has a broad-spectrum antimicrobial activity including against *M. catarrhalis* [42, 43]. Thus, the diminished hBD-2 levels may in part impair both its bactericidal and immunomodulatory action, thereby favoring an inefficient innate pulmonary defense [44]. It is worth adding that one of the strategies of *M. catarrhalis* to colonize the pulmonary epithelium is negative regulation of the inflammatory response of this tissue. It was previously reported that *M. catarrhalis* initiates mostly Toll-like receptor 2- (TLR2-) dependent inflammation of the respiratory epithelium including A549 cells [45]. Furthermore, *M. catarrhalis* diminishes A549 inflammation through engagement of the CEACAM1-binding motif using bacterial surface ubiquitous protein UspA1—which in turn abrogates the TLR2- and NF-*κ*B-triggered inflammatory response [46]. Having confirmed both the presence of UspA1 (ligand for CEACAM1) in OMVs from Mc6 strain [35] and CEACAM1 expression on a certain fraction of A549 cells (this study), our results regarding proinflammatory action of *M. catarrhalis* OMVs do not contradict the previous studies since, (i) studied here, OMVs were potent stimulants only in the presence of IL-1*β* and (2) the diminished, but not abolished, proinflammatory response was documented both for UspA1-containing and UspA1-deficient OMVs [4]. Since changes in the CEACAM1 expression patterns of different fractions of A549 cells can alter their diverse functions [38], the confirmation of CEACAM1 expression is essential in studies on the interaction between *M. catarrhalis* and this line.

Further important insights from this study relied on the demonstration of anti-inflammatory activity of SP and CGRP and their influence on human A549 epithelium cell death and apoptosis. It is important, since excessive cell death including apoptosis together with improper removal of apoptotic cells may contribute to lung inflammation and destruction in emphysema [47, 48]. In the lungs under physiological conditions, epithelial cells have rather low turnover rates (~5%), particularly in the airways [49]. This changes completely following airway epithelial injury [50]. There is limited data demonstrating that some constituents of bacterial OMVs such as OmpA or Omp38 from *Acinetobacter baumannii* may be a potent inducer of macrophage apoptosis and airway epithelial cell apoptosis [51, 52]. Our study revealed that *M. catarrhalis* OMVs induce efficient cell death and apoptosis of A549 cells. Secondly, we documented that physiological concentrations of SP and CGRP can significantly rescue a fraction of A549 cells from OMV-induced apoptosis, indicating important anti-inflammatory potential of both these neuropeptides. Furthermore, since *M. catarrhalis* can induce apoptosis in primary alveolar epithelial cells and A549 cells through engagement of surface protein UspA1 to human CEACAM1 molecule [53], we can hypothesize that OMV-stimulated apoptosis could be at least in part mediated by this surface bacterial protein. Accordingly, as already mentioned, the presence of UspA1 in OMVs from Mc6 strain was confirmed by LC-MS/MS analysis [35]. Here, we have documented the expression of the CEACAM1 receptor on a certain fraction of A549 cells that also supports this hypothesis. It was also in accordance with previous report [38]. On the other hand, our findings reveal that initial postconfluent A549 may be a mixture of CEACAM1-positive and CEACAM1-negative cells. Therefore, the ability of UspA1-containing OMVs to trigger apoptosis of A549 cells with different expressions of CEACAM1 suggests that other factors delivered by OMVs may also be involved in this phenomenon. For example, the involvement of OMV components such as LOS endotoxin in apoptosis was documented previously [54].

The disadvantage attributed to the host through OMV-induced apoptosis is that *M. catarrhalis* triggers cell death that facilitates breaches in the epithelial barrier and therefore assesses to the underlying tissue. Indeed, it was shown that this bacterium can invade bronchial epithelial cells (BEAS-2B), type II pneumocytes (A549), primary small airway epithelial cells (SAEC) [45], and Chang conjunctival cells [55], and it can be effectively hidden inside lymphoid tissue [56]. Therefore, the beneficial antiapoptotic capacity of neuropeptides towards the airway epithelium may prevent the harmful colonization process and severe pathophysiology of downstream infection. The receptor-dependent involvement of both neuropeptides was recently demonstrated in the reduction of primary alveolar epithelial type II cell apoptosis and increased cell survival following oxidative damage [28, 57]. On the other hand, the apoptosis of neutrophils is one of the crucial immunological events which facilitates the resolution of inflammation and prevents tissue damage [11]. Therefore, since *β*-defensins may suppress apoptosis of neutrophils *in vitro* [58] and *in vivo* in COPD patients [13, 59], this study determines that an NP-mediated decrease of hBD-2 may also be advantageous for a relevant apoptotic response.

Because excessive damage to localized tissue is common and unavoidable in many inflammatory disorders, it is highly possible that OMVs and other bacterial stimuli may contribute to this process through degranulation of neutrophils. Therefore, the third aspect of our study focused on whether, and to what extent, CGRP and SP can modulate the intensity of OMV-triggered degranulation of neutrophils. Our previous work demonstrated that CGRP reduced the oxidative burst generated by neutrophils in response to *M. catarrhalis* stimuli [29], contributing potentially to diminished oxidative burden, which in its overwhelming form is responsible for persistence of inflammation [60]. In this study, we report for the first time that *M. catarrhalis* vesicles effectively trigger tissue-destructive degranulation of human neutrophils and that CGRP and SP decrease this process in terms of specific granule release. It indicates that both neuropeptides may potentially reduce granule-dependent harmful effects on surrounding tissue. The unfavorable tissue-destructive as well as airway-remodeling actions of specific granule components such as NGAL in COPD have been documented [12]. Furthermore, since secondary granules are mostly rich in leukocyte adhesion molecules (integrins and adhesive receptor-like proteins), their suppressed release in the presence of neuropeptides may at least, in part, diminish adhesive properties of these cells. Surprisingly, the NP-induced decrease in azurophilic granule exocytosis was not observed. The most likely explanation of this difference is that the physiological concentrations of neuropeptides was too low to visibly affect exocytosis of the most numerous azurophilic granules which as a whole contain the largest proinflammatory content. It was documented that the mobilization of individual granule subsets depends also on the intensity of stimuli. In addition, actin may differentially regulate the exocytosis of each granule [61]. Furthermore, the granules are liberated in a hierarchical fashion in response to various stimuli by first releasing secretory vesicles, followed by tertiary (gelatinase), specific, and finally azurophilic granules [11, 62]. This qualitative grade in granule release minimizes damage whilst preserving functionality. Since the azurophilic and specific granules are the most numerous among four major types present in neutrophils, the impaired degranulation of at least secondary granule contents may be linked to limited proinflammatory mediator release.

## 5. Conclusions

Our study provides novel evidence that SP and CGRP alter the *in vitro* inflammatory milieu of human A549 epithelial cells and neutrophils in response to biologically active *M. catarrhalis* OMVs. Since the reported here OMV-mediated proinflammatory effects may potentially favor the perpetuation of *M. catarrhalis* infection in the lungs, the anti-inflammatory and antiapoptotic activities of SP and CGRP are valuable and may have beneficial implications for reducing this inflammation. The relevance of these findings *in vivo* requires further investigations.

## Figures and Tables

**Table 1:** Percentage of live, apoptotic, and dead A549 cells expressed as means ± SD and analyzed by Annexin V and Dead Cell assay kit using Muse Cell Analyzer.

	Live	Total apoptotic^∗^	Dead
8 h of incubation			
Untreated control	88.42 ± 1.45	10.72 ± 0.99	0.87 ± 0.29
Positive control H_2_O_2_ (5 mM)	67.07 ± 2.60	27.98 ± 4.84	4.93 ± 2.30
OMVs (20 *μ*g/ml)	82.21 ± 0.35	17.70 ± 0.28	0.28 ± 0.18
OMVs (20 *μ*g/ml) + SP (10^−8^ M)	81.64 ± 5.56	17.83 ± 5.06	0.5 ± 0.49
OMVs (20 *μ*g/ml) + CGRP (10^−8^ M)	84.78 ± 0.81	14.90 ± 0.78	0.23 ± 0.18
OMVs (100 *μ*g/ml)	78.28 ± 1.31	20.23 ± 3.01	0.38 ± 0.39
OMVs (100 *μ*g/ml) + SP (10^−8^ M)	82.18 ± 1.45	17.40 ± 1.48	0.43 ± 0.04
OMVs (100 *μ*g/ml) + CGRP (10^−8^ M)	86.00 ± 2.33	13.40 ± 2.19	0.6 ± 0.14
SP (10^−8^ M)	ND	ND	ND
CGRP (10^−8^ M)	ND	ND	ND
24 h of incubation			
Untreated control	86.25 ± 2.14	12.88 ± 1.8	0.87 ± 0.55
Positive control H_2_O_2_ (5 mM)	38.51 ± 0.35	59.99 ± 0.98	1.49 ± 1.34
OMVs (20 *μ*g/ml)	83.98 ± 2.86	15.00 ± 3.04	1.03 ± 0.18
OMVs (20 *μ*g/ml) + SP (10^−8^ M)	90.08 ± 0.60	9.10 ± 0.21	0.83 ± 0.39
OMVs (20 *μ*g/ml) + CGRP (10^−8^ M)	83.33 ± 2.72	15.83 ± 2.72	0.85 ± 0.01
OMVs (100 *μ*g/ml)	68.25 ± 2.18	30.98 ± 2.20	0.70 ± 0.13
OMVs (100 *μ*g/ml) + SP (10^−8^ M)	80.05 ± 0.07	17.80 ± 1.98	2.15 ± 2.05
OMVs (100 *μ*g/ml) + CGRP (10^−8^ M)	83.68 ± 3.08	14.85 ± 1.34	1.48 ± 1.73
SP (10^−8^ M)	82.23 ± 0.46	15.30 ± 1.70	2.48 ± 2.16
CGRP (10^−8^ M)	89.33 ± 1.52	9.43 ± 2.05	1.23 ± 0.53

^∗^Total apoptotic refers to early apoptotic and late apoptotic cells. ND: not determined.

## Data Availability

The genome sequence of Mc6 can be found in GenBank under accession number CP010901.
